# Restrictive state laws aimed at immigrants: Effects on enrollment in the food stamp program by U.S. citizen children in immigrant families

**DOI:** 10.1371/journal.pone.0215327

**Published:** 2019-05-01

**Authors:** Sylvia E. Twersky

**Affiliations:** Health Services Administration and Policy, Temple University College of Public Health, Philadelphia, Pennsylvania, United States of America; Mekong Development Research Institute, VIET NAM

## Abstract

This paper examines whether a chilling effect of restrictive state laws aimed at immigrants creates a barrier to enrollment in the food stamp program (now called the Supplemental Nutrition Assistance Program or SNAP) for U.S. citizen children in low-income immigrant families. This analysis looks at 20 states in the continental United States from 2000 to 2008 that were either at or above the U.S. average for percentage of foreign-born population, or states that ranked in the top 10 percent in terms of change in foreign-born population for that time period. To examine this issue, a multivariate, regression-based difference-in-differences (DD) analysis was applied. The “treatment” group is immigrant families with a U.S. citizen child that is 130% of the federal poverty level or below in states with restrictive immigrant related legislation and the “control” group is native families meeting the same federal poverty level guidelines as well as low-income immigrant families in states without the restrictive legislation. The research findings show that there does not appear to be a chilling effect associated with restrictive state laws on participation in the food stamp program. Food insecurity is an immediate need that may override the impediments to enrollment due to immigration status, causing families to apply despite a negative climate toward immigrants. For policy makers and immigrant advocates it is important to know where chilling effects might not occur in order to work with politicians and federal agencies on crafting sound evidence-based policy. Independent of any chilling effect, the model shows that immigrant families are less likely to enroll in food stamp benefits, consistent with other literature. In addition, independent of the effects of restrictive immigration legislation, both non-citizen and naturalized mothers were less likely to be in a family with food stamp benefits compared to similar native-born mothers. This indicates that all states have a gap in food stamp program enrollment that merits further attention and research.

## Introduction

The Food Stamp (currently called SNAP) program, the largest nutrition assistance program in the U.S., has been shown to address both hunger and food insecurity as well improving general health outcomes for children. The Food Stamp program accomplishes this through both direct provision of food and indirectly, by freeing scarce dollars that can be spent on necessities other than meeting basic food needs. The Food and Nutrition Act of 2008 changed the name of the Federal program from Food Stamps to the Supplemental Nutrition Assistance Program or SNAP as of Oct. 1, 2008. Since during the analysis time frame the program was called the Food Stamp program it will be referred to that way throughout the paper.

The amount of the Food Stamp benefit is based on something called the “Thrifty Food Plan” which was developed to mimic a low-cost healthy diet for different sized households [1]. Under President Kennedy, the first modern pilot program for food stamp benefits was initiated in 1961 and the program became permanent as part of legislation passed in 1964. However, it wasn’t until the 70’s that national eligibility guidelines were established and until 1974 that the program began operating nationwide [1]. The program is administered federally under the U.S. Department of Agriculture (USDA) and provides Electronic Benefit Cards (EBT) to recipients that can be used at grocery stores, supermarkets, farmers’ markets, and other authorized retailers. The Food Stamp program also provides nutrition education.

### Federal and state food stamp policy

#### Eligibility

The federal government fully funds the benefits that recipients receive under the Food Stamp program and has shared responsibility with the states for its administrative costs. The Food Stamp program has both income and asset eligibility tests. States can have an effect on enrollment in terms of administration of the benefits. Federal guidelines for Food Stamps extend benefits up to 130% of the federal poverty (FPL) level for gross income and include an asset test. Net monthly income, after deductions for housing costs and child care, must be less than or equal to 100% of the FPL [2].

As part of the Farm Security and Rural Investment Act of 2002 states gained flexibility in implementation by allowing them to lengthen certification periods, giving states the ability to exclude the value of family cars from the resource test, to expand categorical eligibility if the recipient receives non-cash benefits funded through TANF, and to waive face-to-face recertification. By 2008, there were 12 states that did not require an asset test [2]. In addition, the amount and type of outreach to potential recipients varies by state. By changing these administrative hurdles, states can reduce barriers that impede access to Food Stamps.

#### Benefits

Participants were always expected to cover a portion of food expenses. The Food Stamp Act of 1977 directly tasked beneficiaries with purchasing some of their food stamps. However, this had a depressing effect on program participation and in 1979, participants were given the free portion of their benefits and expected to supplement the stamps with cash (30% of their net income) to reach the full food plan basket. The 1996 Personal Responsibility and Work Opportunity Act (PRWORA) made major changes to the food stamp program including adopting the EBT system, eliminating eligibility for legal non-citizen immigrants, and limiting eligibility for adults without dependents.

In 2000, according to Food Stamp administrative data, there were 17.2 million participants nationwide with an average benefit of $73 dollars per person per month. This monthly average benefit was $184 for households without any elderly. In 2000, slightly over half of all food stamp participants were children [3]. By 2008 there were 28.2 million participants with and average benefit of $102 [4]. The number of children receiving benefits in that year was 13.4 million, about 18% of all children under 18 in the U.S. [5].

#### Food stamp program and outcomes

One effect of Food Stamps is to lift families and children out of poverty. Since most families pool their income and redistribute it among their members, intra-household resource reallocation in response to a SNAP participation could lead to gains in consumption of other goods and services such as housing and other family needs, increasing children’s welfare. The Supplemental Poverty Measure counts food stamps as income, as do multiple other household income measures, and there are numerous studies that show that these benefits are effective in reducing poverty and extreme poverty [6, 7].

Multiple studies have shown that food stamp program participation lowers food insecurity in the household, although the magnitude of the effect varies by study [8–11]. Food insecure households are those that report reduced quality, desirability, and variety of the household diet and there may be multiple indications of disrupted eating patterns and reduced food intake [12]. Using the Panel Survey of Income Dynamics and applying an instrumental variable approach, Mykerezi and Mills [9] estimate that participation in the Food Stamp program reduces by at least 19% the score for household food insecurity. Ratcliffe, McKernan, and Zhang [10] estimate that food stamp benefits reduced the likelihood of being food insecure by about 30% using the Survey of Income and Program Participation (SIPP) data, while Mabli et al. [11] found that household that participated in the Food Stamp program for six months or more showed a decrease in food insecurity by about five to ten percentage points. Looking specifically at immigrant families using the CPS but expanding the scope to all cash benefits, food stamps, and Medicaid, Borjas [13] found that a reduction in the percent of the population that receives these services results in an increase in the percentage of families with food insecurity.

One of the major problems with identifying health outcomes for participants in the food stamp program is selection bias, which makes it difficult to tease out the effects of the program from the fact that children in families that participate in the food stamp program tend to be in poorer health than children in non-participating families. Kreeder, Pepper, Gunderson and Jolliffe [14] account for both selection and measurement errors using NHANES 2001–2006 data and find that the Food Stamp Program improves child health looking at measurements of self-reported health status, obesity, and anemia. Looking at the long-term effects of receiving food stamp benefits in early life (before age 5), Hoynes, Schanzenbach, and Almond [15] find that access to food stamps in childhood leads to a reduction in obesity, high blood pressure, and diabetes in these children as adults. Despite the difficulty in teasing out the effects of the food stamp program there is clear evidence that receipt of food stamps reduces household poverty, decreases food insecurity, and leads to better health outcomes. However, as I discuss in detail below, immigrant families are less likely to receive these benefits compared to native families with similar characteristics.

### Immigrant families and food stamps

According to data from the American Community Survey, children whose parents are foreign born and live in poverty are less likely than children with native parents living in poverty to be enrolled in the food stamp program. This is true whether or not the immigrant parents are U.S. citizens [16]. This data suggests that there are additional barriers that immigrant families face in enrollment compared to native families. According to the 2002 National Survey of America’s Families, the children of immigrants have a significantly higher likelihood of living in a family with one or more food-security problems including running out of food or adults in the family skipping meals [17]. Therefore, children with immigrant parents are at higher risk for food insecurity and hunger but have lower enrollment in the safety net program designed to address these issues.

### Immigrants and eligibility

There are additional eligibility requirements that apply to non-citizen immigrants. Food stamp benefits have never been available to undocumented non-citizens. By contrast, non-citizens who were admitted for humanitarian reasons such as refugees, asylees, and victims of trafficking and battered non-citizens have access to food stamps without a waiting period, as do non-citizen veterans or active duty family members and their families. Prior to PRWORA being passed in 1996, non-citizens legally in the United States were eligible for Food Stamp benefits with the same categorical eligibility as citizens. PRWORA includes the provision that some eligible non-citizens had a maximum benefit of seven years.

The Farm Security and Rural Investment Act of 2002 restored many legal immigrants’ access to food stamp benefits in 2003 including those residing in the US for at least five years, children under 18 regardless of date of entry, and individuals receiving disability benefits. After the 2002 Farm Bill granted eligibility after a five-year waiting period, this eliminated the seven-year time limit. There are seven states that provide nutritional assistance to some or all immigrants that are not eligible under welfare reform legislation (California, Connecticut, Maine, Minnesota, Nebraska, Washington, and Wisconsin) and of those California and Connecticut are part of this analysis. Borjas [13] looked at assistance programs (including cash assistance, food stamps, and Medicaid), and found that food security worsened among immigrant populations after PRWORA passed, a clear indication that access to the social safety net has important implications for health outcomes.

Importantly, an entire family cannot be denied food stamp benefits because the family contains an ineligible immigrant. In addition, since some eligible individuals cannot apply for themselves (for example, US citizen children in a family with non-eligible parents), states are not allowed to require information about the citizenship or eligibility status of any individual not applying for food stamp benefits. States are required to create an application process that allows parents to apply for food stamp benefits for their children without having to disclose their own immigration status. However, one study showed that in some states such as Texas, applications do not clearly state that information such as Social Security Numbers and proof of citizenship was only required for the intended beneficiary. As a consequence, mixed status families may not apply for their eligible children [18].

Each state determines how they calculate income for families with ineligible members. For family members that were never eligible for food stamp benefits, such as undocumented immigrants, states have the option of pro-rating the ineligible members’ share of income or counting the income. For immigrants that became ineligible after PRWORA (i.e. within the five-year waiting period), states have the option of pro-rating the income of the ineligible member or not counting their income and capping the family benefit at a lower amount. The majority of states opt to pro-rate income for all ineligible family members [19].

One important consideration for immigrants and eligibility for Food Stamp benefits is the issue of deeming. When an immigrant enters the U.S. and is sponsored by a family member, it means that the family member signed an affidavit promising to provide enough financial support for that individual to maintain the person at 125 percent of the federal poverty level. This support is required to last until the immigrant being sponsored becomes a US citizen or until they have worked in the U.S. for 40 quarters. This does not apply to children under the age of 18. However, sponsored immigrants are subject to “deeming” when applying for Food stamp benefits for any family members over the age of 18. Deeming is a process in which the state agency counts a portion of the income and assets of the sponsor toward eligibility. Prior to passage of PRWORA, sponsored immigrants were subject to deeming for Food Stamp eligibility for up to three years. After PRWORA deeming is applicable until the immigrant becomes a citizen or meets the work requirements listed above. States may choose to not apply deeming requirements for foods stamp benefits for sponsored immigrant adults. Since children are not subject to deeming, only a portion of a sponsor’s income would be counted for adult family members that are subject to sponsor deeming. This means that the total amount of food stamp benefits for the family may be lowered if an adult in the family is subject to sponsor deeming regulations. There are exceptions to the deeming regulations if the sponsored immigrants are considered indigent [19]. This complicated deeming system may create a climate of fear whereby sponsored immigrants are less likely to apply for food stamp benefits.

Policies related to immigrant eligibility changes over time at the federal level and may be different state by state, as well as different for different social safety net programs within a state. As a result, immigrant families face a high level of complexity and uncertainty in applying for food stamp benefits in a system where the complexity of the application process in general is noted as a barrier for all applicants [20]. In addition, the lack of clarity of the application and uncertainty among applicants about ability of unauthorized immigrants to apply for their U.S. citizen children may create a “chilling effect” where eligible children are not receiving benefits.

## Methodology of analysis

### Treatment and control groups

The purpose of this analysis is to identify whether state laws that target immigrants have any effect on the enrollment of low-income immigrant families with U.S. citizen children in food stamp benefits. An immigrant family is defined as a family where at least one parent is an immigrant, regardless of citizenship status. Looking at the implementation of these state laws as a natural experiment variation in the timing of adoption of these laws across states and over time can be used to identify their impact on family Food Stamp use. The “treatment” group is immigrant families with a U.S. citizen child that is 130% of the federal poverty level or below in states with restrictive immigrant related legislation and the “control” group is native families meeting the same federal poverty level guidelines as well as low-income immigrant families in states without the restrictive legislation. Rather than a person-specific benefit, the Food Stamp Program is a family-level benefit. Therefore, if anyone in the family qualifies for food stamps, the family can receive benefits with the amount based on the number of eligible individuals in the family. The poverty level guidelines reflect a rough estimate of eligibility for food stamp benefits which include a gross income test, a net income test, and an asset test as described above.

A previous analysis of Medicaid [21] showed that living in a state that passed a restrictive law had a small but significant negative effect on enrollment in the year that law was passed, and this negative effect was further mediated by the citizenship of the mother. Some states have passed restrictive legislation, aimed at both undocumented and legal immigrants that target a broad range of activities including obtaining a driver’s license, employment, public education, and access to social safety net programs. The hypothesis is that a chilling effect created by these restrictive laws will be apparent in the uptake of food stamp benefits. A chilling effect is the inhibition of the exercise of legal rights by a perceived threat of some kind of sanction, which in this case applies to U.S. citizen children not accessing food stamp benefits that they are eligible for, because of the restrictive legislation aimed at immigrants. Many immigrant families contain U.S. citizens and both legally present and undocumented individuals within the same family group. In 2000, 19% of children in the U.S. lived in immigrant families and by 2008 this number had jumped to 23% [22]. This chilling effect has previously been demonstrated among immigrants in relation to PRWORA legislation [23–25].

A difference-in-difference approach was used for this analysis. This method eliminates the influence of any unobserved, time-invariant differences between states that adopted and did not adopt restrictive legislation aimed at immigrants that might be correlated both with the adoption of such legislation and the enrollment of families into the food stamp program. In order to further address the possibility of biased estimates, state fixed effects were used to control for unobserved heterogeneity across states and provide estimates of the within-state change in take up of food stamp benefits between eligible immigrant and native families.

The period of 2000 to 2008 was selected so as to avoid confounding any state legislative effects with the earlier implementation of the federal PRWORA legislation noted above. This time period is also consistent with a period of strong growth in state legislation around immigrant issues more generally. Beginning in 1996 with the passage of PRWORA, some states began to fill in the gaps left by the lack of federal coverage for previously covered immigrant groups while others tightened the restrictions further. In 2005, 300 bills dealing with immigration were introduced across all states while 38 laws were enacted. The number of state bills dealing with immigrants continues to increase over time. In 2008 the strong legislative state action around immigration continued with 1,305 state bills considered, of which 206 were enacted [26]. The laws were categorized as falling under regulation, social welfare, or education provisions. The largest category, regulation, included among other things restrictive regulation of employment and access to firearms, and was by the far the type of law most likely to be passed by states during this analysis time period. The second largest category was those laws that address social welfare. However, over half of the laws addressing social welfare passed by states were actually protective in nature, expanding access for certain categories of immigrants. The smallest category of laws involved access to public education and education funding.

The dataset used in this analysis is the March Supplement of the Current Population Survey (CPS). The March supplement is used for research on food stamp program participation because it includes detailed information on income and social safety net program participation [27]. The CPS family data was merged with a state dataset that includes state-specific restrictive immigrant related laws by year and state-specific characteristics by year.

The CPS family data set used in this analysis includes all families that are 130% of the federal poverty line or below in (1) the twenty states at or above the U.S. average for percentage of foreign born population (traditional gateway states), or (2) a state that ranked in the top 10 percent in terms of increase in foreign born population (new destination states) in the time period under consideration (2000 to 2008). This recognizes that states that have a large population of immigrants or states that are experiencing a surge in their immigrant populations may seek to address such increases through legislation that restricts immigrant participation in public programs. States representing criteria one (traditional gateway states) include: Arizona, California, Connecticut, Florida, Illinois, Massachusetts, New Jersey, New York, Rhode Island and Texas. States representing criteria two (new destination states) include: Alabama, Arkansas, Delaware, Georgia, Kentucky, Mississippi, North Carolina, South Carolina and Tennessee. Nevada meets both criteria for inclusion.

### Outcome variables

The outcome variable of interest is whether the family received food stamp benefits. Rather than a person-specific benefit, the Food Stamp Program is a family-level benefit. Therefore, if anyone in the family qualifies for food stamps, the family can receive benefits with the amount based on the number of eligible individuals in the family.

### Family-level predictor variables

Sociodemographic variables include immigrant family indicating whether a family in state s in year t is an immigrant family (value of 1 if one or more parents are immigrants and zero otherwise), citizenship status of mother (citizen, naturalized, non-citizen), family income as a as a percent of the federal poverty level, family race and ethnicity (as indicated by mother’s race and ethnicity), marital status (of mother), number of children in the family, and education level (of mother).

### State-level predictor variables

The following variables are part of this analysis in order to adjust for policy endogeneity, because policymakers are pursuing specific outcomes with the enactment of legislation and therefore the policies cannot be treated as randomly distributed across states. Pre-analysis period state generosity toward immigrants is a constructed variable based on state generosity toward immigrants in 1998, in the wake of welfare reform. The constructed variable is modified from an Urban Institute analysis [28] based on the presence of state-funded programs for immigrants to substitute for Medicaid, the Temporary Assistance for Needy Families **(**TANF), Social Security Income (SSI), and food assistance during the five-year ban period as well as cost sharing and restrictions on these services. The constructed immigrant generosity variable ranks states from 1–4 respectively as least available (1), less available (2), somewhat available (3), and most available (4) for social safety net services for immigrants prior to the analysis period. Additional state characteristics include percent of state residents that are immigrants and the percent of state residents that are not citizens. These variables represent immigrant integration into the community. The percent of state residents over the age of 21 with a high school degree or above indicates community education level. A constructed variable characterizing political party concordance of the state legislature and governor indicates whether they are all republican, all democrat, or mixed. In order to look at state funding available for social safety net programs, state net revenue (this is the difference between state revenues and outlays) is included, although unlike Medicaid the federal government pays for food stamp benefits except for the administrative costs at the state level. The unemployment rate is used as indicator of the economic health of the state and potential need for benefit programs.

### Statistical analysis

To estimate the impact of restrictive state laws, I fit the following linear probability regression model:

$$Y_{hst}=\beta_{0}+\beta_{1}X_{hst}+\beta_{2}Z_{st}+\beta_{3}ResLaw_{st}+\beta_{4}ImmigrantFamily_{hst}+\beta_{5}ResLaw_{st}\times ImmigrantFamily_{hst}+\beta_{6}State_{ht}+\beta_{7}Year_{t}+\epsilon_{hst}$$ (1)

The outcome (Y) will take a value of one if anyone in the family residing in state s in year t is receiving food stamp benefits and is zero otherwise. Household weights are used so that the sampled families reflect the population totals after accounting for any systematic bias in CPS data imputation, and to ensure that estimated effect reflect the behavior of the family population under consideration.

X_*hst*_ is a vector of family characteristics and Z_*st*_ is the vector of state-specific time-varying characteristics. Reslawst takes on a value of 1 if there were any restrictive laws passed in state s at time t, and 0 otherwise. The key variable of interest is the interaction term Reslaw##ImmFam, the product of Reslaw and IMMIGRANTFAMILY. The coefficient on this variable, *β5*, represents the difference-in-differences estimate of the impact of the restrictive law on food stamp enrollment. The analysis was also run for any restrictive laws passed in state s at time t for laws categorized as affecting education, regulation, and as social welfare in order to determine if certain categories of laws had a significant effect on food stamp use.

The control group consists of low-income native families in states that did not pass laws (a 0–0 combination of RESLAW and IMMIGRANTFAMILY), low-income native families in states that passed restrictive laws (1–0 combination of RESLAW and IMMIGRANTFAMILY), and low-income non-native families in states that did not pass laws (0–1 combination of RESLAW and IMMIGRANTFAMILY). This can answer the question of whether non-native families in states that passed restrictive laws were less likely to enroll in food stamp benefits compared to the control group noted above.

State-specific fixed effects, captured by variable STATE, are also included in the model to control for time-invariant differences across all states. However, since the state fixed effects constrain the analysis to the effect of within-state differences, findings were also tested in specifications without these fixed effects to look at differences across states. The within-state comparison (model with a fixed effect) measures the difference between immigrant families’ enrollment in the food stamp program in states that adopted a restrictive immigrant-related law compared to the control group enumerated above. *ε* is a stochastic error term.

This analysis is focusing on binary outcomes (enrolled in food stamps or not enrolled) and nonlinear models such as logit or probit are generally best fitted to analyze such outcomes. However, linear probability models have been used successfully to analyze binary outcomes in the context of difference-in-difference estimation frameworks such as used in this study, particularly in studies looking at insurance market reform and the effect of policy differentials across states, since they provide coefficients that show direct estimates of marginal effects [29–31]. Linear probability models are often preferred with econometric estimation due to their ease of interpretation in natural experiments. In a nonlinear model the coefficient of the DD model’s interaction term cannot be interpreted as a marginal effect and the sign, and magnitude of the coefficient of the interaction term are not accurate, nor is the standard error for these effects [32].

Because citizenship of the mother was shown to have a strong effect on Medicaid/CHIP enrollment among immigrant families in a previous analysis, a second model was used to estimate the effect of citizenship on family enrollment in the food stamp program.

Y_hst=γ_0+γ_1X_hst+γ_2Z_st+γ_3ResLaw_st+γ_4ImmigrantFamily_hst+γ_5ResLaw_st×MothersCitizenship_st+γ_6State_ht+γ_7Year_y+ϵ_hst (2)

Model 2 uses a subset of the sample population, looking at only immigrant families in order to determine if there is an interaction effect of the mother’s citizenship with the passage of restrictive legislation on food stamp program enrollment. In order to address serial correlation which can be an issue in a difference in difference approach [33], both of the logistic regression models were run with cluster-robust standard errors, clustered at the state level.

## Results

### Descriptive statistics

In Table 1, estimates reveal that the participation rates of immigrant and native families in the Food Stamp Program, defined as the number of families participating in the program divided by the number of families that are eligible, varied significantly with 41% of native families and 28% of immigrant families participating in the food stamp program. This is consistent with data that shows that low-income immigrant families are less likely to enroll in the food stamp program compared to similar non-immigrant families. In comparison, at the national level in 2008, the food stamp program served 67% of all eligible individuals [34]. This is up from 59% of all eligible families enrolled in 2000, with a low of 39% in Nevada and a high of 97% participation in Hawaii [35].

**Table 1:** Food stamp participation by family status.

Food Stamp Participation	Immigrant Families	Native Families	Total
No	37.9%	62.1%	24,000,000
Yes	25.3%	74.7%	14,000,000
**Total**	**13,000,000**	**25,000,000**	

Note: Weighted Using CPS household supplement weight

Table 2 provides weighted demographic characteristics of the sample and reveals that the majority of families that receive food stamps are 100% of the federal poverty level or below. Having an unmarried mother with a high school education or below seems more common in food stamp-participating native families. The education between families that participate and don’t participate in the food stamp program seems similar among immigrant families. Overall the education levels among mother’s in immigrant families is lower than among mother’s in native families. Mothers in immigrant families are much more likely to have less than a high school education compared to mothers in native families (60% vs. 32% respectively among families that receive food stamps and 53% vs. 19% among families that do not). The majority of mothers in the immigrant family sample identify as white and Hispanic, while the majority of mothers in native families identify as white non-Hispanic. There is a larger percentage of mothers that identify as Black in native families receiving food stamps.

**Table 2:** Demographic characteristics of families who receive food stamps in the 20 selected states, 2000–2008: Immigrant compared to native.

	Native Families: Food Stamps	Native Families: No Food Stamps	Immigrant Families: Food Stamps	Immigrant Families: No Food Stamps
Total (Weighted):	10,000,000	15,000,000	3,500,000	9,100,000
**Poverty Level**				
100% or Below FPL	86.0%	68.9%	87.6%	66.8%
101–130% FPL	14.0%	31.1%	12.4%	33.3%
**Mother Hispanic**				
Not Hispanic	85.2%	85.1%	19.7%	22.7%
Hispanic	11.2%	11.2%	68.5%	73.3%
Puerto Rican	3.1%	2.2%	11.8%	4.0%
**Marital Status of Mother**				
Married	21.0%	38.8%	40.5%	69.6%
Married-Spouse Absent	2.5%	2.3%	3.8%	2.8%
Not Married	76.5%	58.9%	55.7%	27.7%
**Education of Mother**				
Less than High School	31.9%	18.4%	59.9%	52.6%
High School Grad	41.2%	42.4%	25.7%	28.4%
Some College	24.9%	31.0%	11.6%	12.6%
College Graduate	2.0%	8.3%	2.8%	6.4%
**Race of Mother**				
White	54.6%	71.4%	84.1%	84.1%
Black	43.0%	26.3%	9.7%	7.0%
American Indian	2.2%	1.9%	1.3%	1.6%
Asian	.2%	.5%	4.9%	7.4%
**Num. of Own Children (Mother)**				
1	24.5%	31.8%	15.8%	21.6%
2	33.1%	35.1%	29.1%	34.0%
3	25.4%	21.4%	29.2%	27.0%
4	10.8%	7.8%	15.7%	11.8%
5+	5.3%	4.1%	10.2%	5.5%

Note: Weights are the household weights for the CPS March supplement

### Regression results

#### Outcomes

The results of the linear probability regression models in Table 3 indicate that there is no chilling effect observable among immigrant families in states that pass restrictive legislation. None of the interaction terms between immigrant law and family type are statistically significant for either model 1, the interaction between immigrant families and the presence of restrictive legislation, or model 2, the interaction of the mother’s citizenship and restrictive legislation within the subsample of all immigrant families. This holds true for findings in Table 4 when the models include or exclude state fixed effects (i.e., models yielding within-state estimates and across-state estimates, respectively). This also holds true for the sensitivity analysis discussed below and found in Table 5. There is no evidence of a chilling effect of restrictive immigrant-related legislation on food stamp enrollment among immigrant families. However, there is an independent and statistically significant effect of being in an immigrant family, which makes those families four percentage points less likely to enroll in food stamps compared to native families (model 1). This is consistent with the literature, which shows that immigrant families are less likely to enroll in food stamp benefits [36–38].

**Table 3:** Full Model: Effect of presence of restrictive state laws on low income families’ uptake of food stamp benefits: 2000–2008.

20 State Analysis	All Laws, All Families	All Laws, Immigrant Families	Only Social Welfare Laws, All Families	Only Social Welfare Laws, Immigrant Families
Restrictive Law	0.007 (.0133)	0.011 (.0189)	-0.013 (.0104)	-0.034 (.0297)
Law*Immigrant Family	0.002 (.0204)	NA	0.012 (.0173)	NA
Law*Non-Citizen Mother	NA	.000 (.0254)	NA	0.028 (.0212)
Law*Naturalized Mom	NA	-.010 (.0198)	NA	-0.003 (.0281)
Immigrant Family	-0.040*** (.0126)	NA	-0.058 *** (.0097)	NA
Citizenship of Mother				
Naturalized	-0.099*** (.0149)	-0.083*** (.0199)	-0.109*** (.0166)	-0.087*** (.0206)
Not a Citizen	-0.085*** (.0113)	-0.056*** (.0149)	-0.117*** (.0095)	-0.060*** (.0121)
Poverty Level				
101–130% of FPL	-0.192*** (.0132)	-0.172*** (.0158)	-0.225*** (.0127)	-0.172*** (.0159)
Hispanic Mother
Hispanic	0.024 (.0121)	0.020 (.0261)	0.032 (.0282)	0.019 (.0261)
Puerto Rican	0.104*** (.0208)	0.130*** (.0350)	0.138*** (.0215)	0.127*** (.0356)
Marital status of Mother				
Married-Spouse Absent	0.117*** (.0266)	0.118*** (.0295)	0.117*** (.0262)	0.117*** (.0295)
Not Married	0.171*** (.0156)	0.210*** (.0172)	0.171*** (.0156)	0.210*** (.0173)
Education of Mother				
High school Grad	-0.077*** (.0075)	-0.040** (.0187)	-0.087*** (.0103)	-0.040** (.0187)
Some College	-0.114*** (.0147)	-0.046** (.0215)	-0.121`*** (.0168)	-0.047** (.0214)
College Graduate	-0.242 (.0148)	-0.113*** (.0113)	-0.278*** (.0150)	-0.114*** (.0114)
Race of Mother				
Black	0.110*** (.0088)	0.005 (.0157)	0.161*** (.0095)	0.005 (.0158)
American Indian	0.020 (.0312)	-0.032 (.0381)	0.034 (.0345)	-0.031 (.0387)
Asian	0.019 (.0520)	-0.001 (.0520)	0.017 (.0574)	-0.002 (.0518)
Other	-0.120 (.0749)	-0.073*** (.0158)	-0.194** (.0742)	-0.079*** (.0181)
Number of Children (Mother)	0.046*** (.0026)	0.048*** (.0034)	0.046*** (.0026)	0.048*** (.0034)
State Unemployment Rate	0.031*** (.0026)	0.024* (.0141)	0.030*** (.0086)	0.024* (.0126)
% of State Pop. Immigrants	-0.004 (.0139)	0.008 (.0208)	.000 (.0142)	0.014 (.0189)
% of State Non-Citizen Immigrants	0.005* (.0026)	0.002 (.0055)	0.004 (.0026)	0.002 (.0054)
% of State HS Grad. and above	0.017** (.0073)	0.004 (.0136)	0.019** (.0077)	0.004 (.0127)
State Net Revenue	-2.31e-07 (1.38e-06)		2.89e-07 (1.10e-06)	9.03e-07 (1.61e-06)
State Gov. Party Concordance				
All Republican	-0.031 (.0236)	-0.032 (.0462)	-0.023 (.0240)	-0.025 (.0482)
Mixed	-0.034* (.0179)	-0.049*** (.0178)	-0.038* (.0198)	-0.046** (.0173)
Pre-Analysis State Generosity				
Less Available	-0.049 (.1651)	0.074 (.2595)	-0.006 (.1689)	0.167 (.2486)
Somewhat Available	0.022 (.0970)	0.013 (.1972)	-0.123 (.0946)	-0.031 (.2051)
Most Available	-0.010 (.1542)	-0.146 (.2675)	-0.053 (.1557)	-0.219 (.2595)

*p ≤ .1

**p ≤ .05

***p ≤ .01

Notes: All Families include those with a family income 130% or below of federal poverty level. Immigrant Families at least one non-native parent with family income 130% or below of federal poverty level. Social Welfare law only includes state measures that further restrict access to means-tested programs based on immigrant status. State and year fixed effects were included. In this linear probability model, data was weighted and the standard error was clustered at the state level.

**Table 4:** Effect of restrictive laws with and without state fixed effects.

20 State Analysis	All Laws Within State	All Laws Across States	Only Social Welfare Laws Within State	Only Social Welfare Laws Across States
**All Families**				
Restrictive Law	0.007 (.0133)	0.001 (.0144)	-0.021 (.0123)	-0.013 (.0058)
Law*Immigrant Family	0.002 (.0204)	0.010 (.0200)	0.005 (.0148)	0.018 (.0158)
Immigrant family	-0.040*** (.0126)	-.0410*** (.0127)	-0.038 *** (.0107)	-0.061*** (.0103)
**Immigrant Families**				
Restrictive Law	0.011 (.0189)	0.011 (.0224)	-.029 (.0302)	-0.034 (.0297
Law*Non-Citizen Mother	.000 (.0254)	0.005 (.0258)	.027 (.0220)	0.028 (.0212)
Law*Naturalized Mom	-.010 (.0198)	-0.008 (.0196)	-0.009 (.0293)	-0.003 (.0281)

*p ≤ .1

**p ≤ .05

***p ≤ .01

Notes: All Families include those with a family income 130% or below of federal poverty level. Immigrant Families at least one non-native parent with family income 130% or below of federal poverty level. Social Welfare law only includes state measures that further restrict access to means-tested programs based on immigrant status. This was included as it was the only law subset that proved to be significant. In this linear probability model, data was weighted and the standard error was clustered at the state level. Year fixed effects were used. Regression controlled for: mother’s citizenship, race, ethnicity, number of children, and education; family poverty level; State characteristics including: Unemployment rate, % of State Pop. Immigrants, % of State Non-Citizen Immigrants, % of State HS Grad. and above, State Net Revenue, State Gov. Party Concordance and Pre-Analysis State Generosity.

**Table 5:** Sensitivity Tests- Low income families uptake of food stamp program, 2000–2008.

20 State Analysis	All Families	Families with One Child	Families With Siblings	Two-parent Families	One-parent Families
Restrictive Law	0.007 (.0133)	0.009 (.0158)	0.003 (.0157)	0.015 (.0158)	0.000 (.0175)
RestrictiveLaw*Immigrant Family	0.002 (.0204)	0.012 (.0291)	0.003 (.0243)	0.003 (.0169)	-0.002 (.0329)
Immigrant Family	-0.040*** (.0126)	-0.030 (.0416)	-0.047*** (.0125)	-0.037*** (.0124)	-0.043* (.0219)
SocialWelfare Law	-0.013 (.0058)	-0.008 (.0115)	-0.022*** (.0068)	-0.011 (.0160)	-0.017 (.0181)
SocialWelfare*Immigrant Family	0.018 (.0158)	-0.035 (.0272)	0.015 (.0201)	-0.024 (.0336)	.0408 (.0313)
Immigrant Family	-0.061 *** (.0103)	-0.036 (.0335)	-0.072*** (.0126)	-0.032*** (.0114)	-0.043** (.0170)

*p ≤ .1

**p ≤ .05

***p ≤ .01

Notes: All Families include those with a family income 130% or below of federal poverty level. Immigrant Families at least one non-native parent with family income 130% or below of federal poverty level. Social Welfare law only includes state measures that further restrict access to means-tested programs based on immigrant status. In this linear probability model, data was weighted and the standard error was clustered at the state level. State and year fixed effects were used. Regression controlled for: mother’s citizenship, race, ethnicity, number of children, and education; family poverty level; State characteristics including: Unemployment rate, % of State Pop. Immigrants, % of State Non-Citizen Immigrants, % of State HS Grad. and above, State Net Revenue, State Gov. Party Concordance and Pre-Analysis State Generosity

#### Sociodemographic characteristics

Looking at model 1, eligible immigrant families are four percentage points less likely than eligible native families to participate in the food stamp program. Having a mother that is a naturalized citizen or a non-citizen makes the family 9.9 percentage points and 8.5 percentage points, respectively, less likely to participate in the food stamp program compared to families where the mother is a U.S. native. The magnitude of this effect is slightly reduced when the analysis is restricted to immigrant families, with an 8.3 percentage point reduction for mothers who are naturalized citizen and a 5.5 percentage point reduction for non-citizen mothers, respectively. As indicated in the descriptive data, families that are 100–130% of the poverty level are 19.2 percentage points less likely to participate in the Food Stamp Program compared to families that are 100% of the federal poverty level or below.

Puerto Rican mothers are significantly more likely to participate in the food stamp program compared to white mothers, and this effect is slightly larger in immigrant families (10.4 percentage points compared to 13 percentage points). Single-parent families are more likely to enroll in food stamps than married families, with a strong effect seen for non-married families (11.7 percentage point decrease in food stamp participation for married families with a spouse absent; 17.1 percentage point decrease in enrollment for not married compared to married families). Education of the mother is significantly and inversely related to likelihood of food stamp participation, with increasing educational attainment decreasing the likelihood of being enrolled compared to mothers with less than a high school education. The only racial group with a significant association with food stamp participation is among Black mothers who are 11 percentage points more likely to be enrolled than white mothers. Having an additional child in the family is associated with a 4.5 percentage point increase in enrollment.

#### State level characteristics

Again, looking at model 1, increases in the state unemployment rate are associated with an increase in the likelihood of a family being enrolled in the food stamp. For the full family sample, but not the sub- sample of immigrant families, as the percent of the state population with a high school diploma or greater increases there is a very small but significant increase in enrollment in food stamps. Only the immigrant family sub-sample shows a significant effect of the political composition of state governments on participation in the food stamp program. In states and years with a mix of Republican and Democratic governance between the governor’s office and the state legislature there is a significant decrease in food stamp participation (by 4.9 percentage points) compared to an all democratic party state governance structure. Interestingly there is no effect seen for all Republican party state governance.

### Sensitivity tests

Two states among the 20 chosen, California and Connecticut, are potentially influential outliers since these are among seven states in the U.S. that provide state-only food benefits for immigrants that are ineligible for the federal Food Stamp Program. Thus, they were excluded from the analysis as part of a sensitivity test to see if the state level generosity was potentially changing uptake of the food stamp program. There were no differences in the outcomes of interest (interaction between restrictive laws and immigrant families) excluding these two states, so they were left in the final model.

Other sensitivity tests to address the robustness of the model included looking at a sub-sample that includes only immigrant families (one or both parents foreign born) looking at the interaction effect of restrictive laws and citizenship status of the mother, running the models with single-parent families vs. two-parent families, and comparing models for families with one child vs. families with multiple children. See Tables 2 (immigrant family subset) and 3 (different family compositions) for results of the sensitivity analysis. None of these tests revealed any impact of the restrictive laws on immigrant food stamp participation.

### Limitations

There are often unobserved fluctuations during the year in income among families and therefore there may be some families that are eligible part of the year for food stamps that have annual incomes above 130% of the federal poverty level. In one analysis using the Panel Study of Income Dynamics almost half of families that reported having federal food stamp benefits had annual incomes above 130% of the federal poverty level [9]. Therefore, the safe cutoff of 130% of the federal poverty level excluded some families that may have been eligible to receive food stamp benefits.

In addition, the eligibility requirements for the food stamp program can be difficult and burdensome, with some states having asset tests that include the value of the family’s car(s) and requiring re-certification every few months. Ideally it would be possible to monitor fluctuations in income and eligibility as well specific eligibility requirements by state. However, the CPS is commonly used to look at enrollment in food stamp benefits (12 month look back period) and multiple studies use presumptive eligibility as this study does. The outcomes are consistent with existing literature, so the analysis was not negatively affected by using presumptive eligibility based on family income as a percent of the federal poverty line.

There is also an issue of misreporting bias when it comes to large population studies and reporting on cash benefits. Looking at Texas, Maryland and Illinois food stamp administrative program data compared to the 2006 March supplement of the CPS, Parker [39] found that 50% of the households receiving these benefits did not report receipt. Mayer and George [40] also found that 50% of families are not reporting receipt of food stamp benefits in the CPS. However, the benefits of taking a difference in differences approach is that as long as the misreporting of receipt of CPS is not different for immigrant and native families (and there is no research to suggest that it is) then this misreporting bias should not affect the outcome of interest for this paper.

## Discussion

The mean value of food stamps is different between immigrant and native families. The average weighted mean for immigrant families is $750 for the year, while for native families it is $1125 for the year. This may be due to the fact that immigrant families have one or more family members who are not eligible for benefits due to their immigration status. However, this could be a driver for the difference in enrollment between immigrant and native families since those eligible for higher benefits tend to participate at higher rates than other eligible individuals [41]. This idea is supported by the fact that having a larger number of children, even holding income as a percent of the federal poverty line constant, significantly increases the likelihood of participating in the food stamp program. Since additional benefits are paid for each eligible family member, it may be that the incentive to participate in the program increases as the benefit dollars increase. In addition, larger family size may reflect greater need for the non-cash support.

There does not appear to be a chilling effect associated with restrictive state laws on participation in the food stamp program. Some research related to implementation of PRWORA considered the potential chilling effect on food stamp benefits and found that increased food insecurity among children of non-citizens was due to changes in program participation rules that reduced their benefit amount rather than by reducing participation among immigrant families [42]. This may reflect a few key facts about the program. Potentially food insecurity is an immediate need that overrides the chilling effect, causing families to apply despite a negative immigration climate. Another explanation is that while benefits are reduced by the number of ineligible family members, this benefit can be used to support the food needs of the entire family, making participation more attractive even for mixed status families.

One argument that was made against the chilling effect of PRWORA on food stamp participation among immigrant families is that much of this can be explained by the naturalization of these immigrants as a response to welfare reform [43]. In order to address this concern, families were classified as immigrant families based on the parent’s nativity and not on citizenship status. In addition, the analysis specifically looks at naturalized immigrants separate from both non-citizens and naturalized citizens and finds that naturalized citizens are still significantly less likely to participate in the food stamp program compared to native citizens. This may be due to requirements in the process of naturalization that discourage safety net benefit use or may reflect the fact that a family with a naturalized citizen may be a mixed status family, where one or more members may not qualify for participation in food stamps due to immigration status.

Skinner [44] looked at SNAP take-up in 19 states among immigrant families with children. Using the American Community Survey (ACS) data to determine SNAP program enrollment and socioeconomic characteristics of the children for his analysis he draws on legislative data from 2005 through 2008 creating a general variable that labels the characteristic of the state neutral, expansive or restrictive. Skinner finds marginal evidence that states that he categorized as restrictive are associated with a reduction in SNAP enrollment among immigrant families with at least one citizen child (p ≤ 0.10). The marginal statistical significance and his lack of established criteria for classifying laws may account for the difference in results between Skinner’s findings and this analysis.

This study’s approach of looking at health in all policies following CDC and WHO guidelines [45] allows policy makers and advocates to assess the potential chilling effect of laws that include both direct effects on social safety net programs such as food stamp benefits as well as laws that may have an indirect effect (such as laws that regulate drivers licenses, job access for immigrants, and access to education). This study was adequately powered to detect effects of a policy relevant magnitude, and a previous study looking at Medicaid receipt in this population did find a significant chilling effect of these laws [21]. The fact that immigrant families are less likely to participate in the food stamp program but that a chilling effect is not the driver behind this difference in participation is key information for both law makers and immigrant advocates. This can provide a potential focus for increasing enrollment among at risk and eligible immigrant families.

The sociodemographic factors that were found to be significant in increasing or decreasing food stamp program participation were all reflective of the wider literature, which shows that independent of any restrictive laws immigrant families are less likely to participate than native families, Black families are more likely to participate than white families, and that lower income level increases participation even within the income eligibility criteria [36–38].

While a state level chilling effect of anti-immigrant restrictive legislation was not seen, independent of state laws immigrant families were less likely to enroll in food stamp benefits compared to their native counterparts. In addition, independent of the effects of restrictive immigration legislation, both non-citizen and naturalized mothers were less likely to be in a family with food stamp benefits compared to similar native mothers. This research (consistent with the general literature) still suggests that immigrants are an important target group for SNAP enrollment because of lower numbers of participation and higher needs than native families.
